# Successful desensitization to atezolizumab-induced near-fatal anaphylaxis in patients with hepatocellular carcinoma: A case report and literature review

**DOI:** 10.5415/apallergy.0000000000000138

**Published:** 2024-04-04

**Authors:** Ji Hyun Oh, Kwang Il Seo, Hee-Kyoo Kim, Gil-Soon Choi

**Affiliations:** 1 Department of Internal Medicine, Kosin University College of Medicine, Busan, Republic of Korea

**Keywords:** Anaphylaxis, atezolizumab, desensitization, hypersensitivity

## Abstract

Atezolizumab, a humanized antiprogrammed death ligand 1 monoclonal immunoglobulin G1 antibody, is a targeted therapeutic drug known as an immune checkpoint inhibitor. It is currently used to treat various types of cancer, including unresectable hepatocellular carcinoma (HCC), nonsmall cell lung cancer, urothelial cancer, and breast cancer, and is becoming a therapeutic option in the forefront of oncology treatment. However, it may sometimes lead to undesirable adverse reactions owing to the activation of immune responses in various organs. Cutaneous adverse reactions to atezolizumab are well known; however, cases of anaphylaxis are very rare. In this report, we present the first case of HCC who experienced near-fatal anaphylaxis to atezolizumab in South Korea.

## 1. Introduction

Atezolizumab, a type of targeted therapy drug known as an immune checkpoint inhibitor (ICI), is a humanized monoclonal immunoglobulin G1 antibody that binds to programmed death ligand 1 (PD-L1) and inhibits this signal pathway, thereby causing tumor-specific cytotoxic T-cell immunity [1, 2]. It is currently used in the treatment of various advanced cancers, including unresectable hepatocellular carcinoma (HCC), nonsmall cell lung cancer, breast cancer, and urothelial cancer [1, 3]. Although it is becoming a therapeutic option in frontier fields of oncology, it can sometimes lead to adverse reactions resulting from the activation of immune responses in various organs. Skin-related hypersensitivity reactions (HSRs) to atezolizumab are well documented, with more than one-third of the patients treated in clinical trials experiencing skin reactions [4]. However, anaphylaxis was rare. Here, we describe a case of near-fatal anaphylaxis induced by atezolizumab in a 69-year-old man who was undergoing chemotherapy for unresectable HCC.

## 2. Case presentation

A 69-year-old man was diagnosed with HCC with lung metastasis. After a multidisciplinary discussion, it was decided that the patient would receive chemotherapy combining atezolizumab (1,200 mg) and bevacizumab. Five minutes after starting the first cycle of atezolizumab, he presented with facial flushing and generalized itching and soon lost consciousness with hypotension and an oxygen saturation of 90%. Atezolizumab was immediately discontinued, and the patient was treated with intravenous dexamethasone and chlorpheniramine. Norepinephrine fluid was also used to increase blood pressure. However, even after 15 minutes, his condition did not improve and he was transferred to the intensive care unit for close observation and further treatment, followed by referral to our allergy clinic. Due to suspected anaphylaxis, intramuscular epinephrine was immediately injected, leading to gradual improvement in his condition, and blood samplings for measuring the serum tryptase level were done. After this event improved, bevacizumab was administered according to scheduled protocols, and there were no adverse reactions.

One week after discharge, the patient visited the outpatient allergic clinic. The serum tryptase level during the event was elevated to 36.5 ng/mL, and follow-up tryptase level was within the normal range. The patient was confirmed as atezolizumab-induced anaphylaxis. For further evaluation, skin test for atezolizumab was recommended, but the patient refused it due to cost concerns. The patient had no history of allergies, but his clinical symptoms were suggestive of asthma. Pulmonary function tests were performed, which confirmed the diagnosis of asthma. The patient was finally diagnosed with HCC with lung metastasis, atezolizumab-induced anaphylaxis, and bronchial asthma. For persistent administration of atezolizumab in HCC treatment, desensitization therapy was considered; however, the patient declined further administration because of the potential for recurrent HSRs.

After 3 weeks, the patient was readmitted for a regimen change and follow-up magnetic resonance imaging, which showed a significant improvement in HCC. Therefore, we decided to continue the previous treatment without changing the regimen, and desensitization to atezolizumab was planned. Owing to the severity of the symptoms, the desensitization protocol was planned using 2-bag 17 steps (Table 1), which is a modification of the 1-bag desensitization protocol. Premedication consisting of antihistamines, leukotriene receptor blockers, and systemic steroids was administered. Fluids with a normal saline solution were used at 40 mL/h throughout the first 9 steps. The patient experienced generalized urticaria with itching at the 16th step of the first desensitization. However, he completed the treatment without a breakthrough reaction from 2nd desensitization without any changes in the premedication and desensitization protocols.

**Table 1.** Desensitization protocol

Step	Solution	Rate (mL/h)	Time (min)	Volume/step (mL)	Administered dose (mg)	Cumulative dose (mg)
1	B	0.2	15	0.05	0.022	0.022
2	B	0.6	15	0.15	0.067	0.089
3	B	1.8	15	0.45	0.200	0.289
4	B	2.6	15	0.65	0.289	0.577
5	B	5.2	15	1.30	0.577	1.154
6	B	10	15	2.50	1.110	2.264
7	B	15	15	3.75	1.665	3.929
8	B	22.5	15	5.63	2.498	6.427
9	B	33.7	15	8.43	3.741	10.168
10	B	50.5	15	12.63	5.606	15.773
11	B	75.7	15	18.93	8.403	24.176
12	B	113.5	15	28.38	12.599	36.774
13	A	10	15	2.50	11.1	47.874
14	A	20	15	5.00	22.2	70.074
15	A	35	15	8.75	38.85	108.924
16	A	50	15	12.50	55.5	164.424
17	A	70	198.2	231.25	1026.75	1191.174

Solution preparation: solution A, 1:1 solution 4.444 mg/mL, 260 mL; solution B, 1:10 solution 0.444 mg/mL, 100 mL.

## 3. Discussion

Although atezolizumab, a representative ICI drug, induces immune activation with strong antitumor effects, it can lead to various undesirable immune-related adverse reactions, including skin, gastrointestinal, endocrine, and liver reactions [1]. According to the European Medicines Agency 2019 assessment report, HSRs of atezolizumab have been reported in up to 10% of patients, and the recent BC Cancer Agency Drug Manual suggests HSRs in ~18% of patients [5-7]. Although cutaneous HSRs, such as rash and pruritus, are common, anaphylaxis induced by atezolizumab is considered rare even among ICI drugs. Based on the literature review, 3 cases of atezolizumab-induced anaphylaxis were documented (Table 2). This report presents the first case of near-fatal anaphylaxis induced by atezolizumab treatment in this country. Our patient developed severe anaphylaxis during the first administration of atezolizumab, which was successfully managed with desensitization therapy, despite experiencing near-fatal anaphylaxis.

**Table 2.** Reported cases of atezolizumab-induced anaphylaxis

	Gonzalez-Diaz et al.[8]	Zhao et al.[9]	Bian et al.[10]	Our case
Case number	1	1	1	1
Age/gender	64/male	64/male	75/male	69/male
Underlying disease	Metastatic lung cancer	Recurred small cell lung cancer	Recurred HCC s/p right radical hepatectomy	Unresectable HCC
Route of administration	Intravenous	Intravenous	Intravenous	Intravenous
Symptoms	Chest pain, diaphoresis, dizziness, and hypotension	Dyspnea, palpitations, chest tightness, a general prickling sensation, cold limbs, cyanosis, hypotension, and LOC	Dyspnea, dizziness, numbness in his feet, hypotension, and LOC	Facial flushing, generalized itching, hypotension, oxygen saturation of 90%, and LOC
Severity grade	IV	IV	IV	IV
Reaction occurrence time	30 min	3 min	10 min	5 min
Number of exposures at the time of occurrence	Second	Second*	First	First
Allergic evaluation	Negative in prick test, positive in intradermal test	NA	NA	NA
Serum tryptase level	NA	NA	NA	Elevated
Desensitization	Yes (4 bags 16 steps protocol)	No (regimen changed)	No (regimen changed)	Yes (2 bags 17 steps protocol)

HCC, hepatocellular carcinoma; IV, intravenous; LOC, loss of consciousness; NA, not available.

* He had a grade 2 rash in first administration of atezolizumab

Anaphylaxis is defined as “a serious, generalized or systemic, allergic or HSR that can be life-threatening or fatal” [11]. The disease can progress rapidly and lead to death. Therefore, it is crucial to quickly recognize this condition and provide appropriate treatment. Nevertheless, underdiagnosis and undertreatment of anaphylaxis have been shown to be common in the real world [12, 13]. Anaphylaxis due to general causes is reported to exhibit mucocutaneous symptoms in 80% of cases; however, in cases of drug-induced anaphylaxis, only 30% are accompanied by skin symptoms, making diagnosis relatively challenging [12, 14]. Besides, anaphylaxis may not be clinically distinguishable from infusion reactions (IRs) during chemotherapy. In this patient, the HSRs occurred immediately after administration of atezolizumab on its first administration, which was initially mistaken for IRs. Serum tryptase can aid in differentiating anaphylaxis from its mimics [14]. An increase in serum tryptase levels indicates mast cell/basophil degranulation and suggests a mast cell/basophil-mediated anaphylactic reaction, whether IgE mediated or non-IgE mediated. In the present case, the tryptase level during the event was elevated, supporting the diagnosis of anaphylaxis. According to the Food and Drug Administration 2016 label, severe IRs of atezolizumab were observed in 1.3% to 1.7% of patients [4], suggesting that the frequency of atezolizumab-induced anaphylaxis may be higher than that reported. Therefore, physicians should be aware of possibility of anaphylaxis due to atezolizumab and be highly alert about its occurrence.

Emergency treatment for anaphylaxis is required immediately to avoid treatment delays. After assessment of respiration and circulation, infusion of the causative agent is stopped immediately, and the first-line treatment is an intramuscular injection of epinephrine [14, 15]. If the patient shows no response to epinephrine within 5 to 10 minutes, epinephrine administration should be repeated. Delayed administration of epinephrine is known to be one of the risk factors for serious and fatal anaphylaxis [16]. Therefore, immediate administration of epinephrine is important as soon as anaphylaxis is recognized.

Anaphylaxis is traditionally thought to be mediated by IgE, with mast cell and basophils as key cells. However, mast cells and basophils can also be activated through alternative mechanism such as IgG antigen interaction, MRGPRX2 transmembrane protein, modulation of kinin system, and complement system through anaphylatoxin (C3a, C5a, and C5b-9), all of which can lead to anaphylaxis [17-19]. Anaphylaxis can also be caused by cytokine release syndrome [18]. The phenotypes and endotypes of anaphylaxis can be further assessed through skin test, specific IgE test, and basophil activation test (BAT), which help identify mast cell involvements in the HSR as well as cross-reactivity between drugs [19]. The mechanisms underlying atezolizumab-induced anaphylaxis remain unclear. One study reported a positive reaction in an intradermal skin test with atezolizumab that was related to IgE-mediated reactions [8]. It has been also suggested that anaphylaxis may be due to the suppression of PD-L1, which led to the enhancement of the patient’s T-cell immune function and thereby increased the production of high-affinity IgE [9]. Besides, anaphylaxis due to atezolizumab may be IgG mediated, as has been suggested with other biologic and chemotherapeutic agents [17]. In the present case, although we did not perform skin test or BAT, serum tryptase level was elevated, indicating the mast cell activation. Bian et al. [10] reported a patient who experienced anaphylaxis during the first injection of atezolizumab. Similarly, our case involved anaphylaxis after the initial exposure of atezolizumab, suggesting that anaphylaxis occurred without sensitization to allergen. These findings indicate that atezolizumab-induced anaphylaxis may be caused by mast cell activation through non-IgE other mechanism than IgE mediated. Further studies are required to elucidate the underlying mechanisms. Rarely, anaphylaxis may result from inactive ingredients included in drug. All ICIs contain polysorbate 20 or polysorbate 80 [17]. If a patient experiences anaphylaxis with multiple drugs, the excipients, rather than the drug itself, are also considered as a potential cause of anaphylaxis, although the likelihood is low.

If atezolizumab is the first-line treatment option or if it is more effective than other medications, desensitization can be considered under the supervision of an experienced allergist. Desensitization therapy is that patients receive medication in incremental doses starting from a small amount and ultimately reaching the target dose [20]. It was indicated for patients with immediate reactions induced by mast cell/IgE and non-IgE activation and with certain delayed type IV reactions [19]. In this case, even though the patient experienced near-fatal anaphylaxis, the effectiveness of atezolizumab was deemed significant; therefore, desensitization treatment was considered. A commonly used desensitization protocol involves the use of stepwise diluted solutions, such as a 3-bag 12-step or 4-bag 16-step protocol. Limited information is available regarding desensitization to atezolizumab in patients with serious HSR. Gonzalez-Diaz et al. [8] reported that the 4-bag 16-step desensitization protocol for atezolizumab and bevacizumab was useful after severe anaphylaxis in the treatment of lung adenocarcinoma. In Korea, 1-bag protocols without dilution have been recently reported, with the advantages of reduced time and the risk of potential errors without compromising the safety, efficacy, and tolerability of the process [21, 22]. In the present case, a 1-bag protocol was initially considered; however, a modified 2-bag approach was used due to the severity of the patient’s previous reaction. Skin test not only helps identify the causative drug and understand reaction mechanism but is also useful in determining the starting concentration of desensitization and predicting the risk of breakthrough reaction during desensitization [19]. In this case, skin test was not performed, which did not influence the decision to proceed with desensitization. Even if the skin test result was negative, the management remained unchanged. The patient completed the atezolizumab injection with desensitization. Our case demonstrates that a desensitization protocol using 2-bag 17 steps can be clinically applied for severe anaphylaxis.

## 4. Conclusion

This is the first case report of atezolizumab-induced anaphylaxis in South Korea, showing successful administration through desensitization. Although the frequency of anaphylactic shock caused by atezolizumab treatment is extremely low, physicians should be aware of this possibility and should be highly alert to the occurrence of severe anaphylaxis. Further studies are needed to understand the underlying mechanisms of HSRs and establish desensitization protocols.

## Acknowledgements

The authors wish to be grateful to our patient. The patient provided consent for publication.

## Conflicts of interest

The authors declare no conflicts of interest.

## Author contributions

Conceptualization: Oh JH and Choi GS. Data curation: Oh JH, Seo KI, and Choi GS. Treatment: Seo KI and Choi GS. Investigation: Oh JH and Choi GS. Visualization: Oh JH and Choi GS. Writing original draft: Oh JH and Choi GS. Writing review and editing: Choi GS, Seo KI, Oh JH, and Kim HK. Final version: all approved.
